# Toward core inter-professional health promotion competencies to address the non-communicable diseases and their risk factors through knowledge translation: Curriculum content assessment

**DOI:** 10.1186/1471-2458-14-717

**Published:** 2014-07-14

**Authors:** Elizabeth Dean, Marilyn Moffat, Margot Skinner, Armele Dornelas de Andrade, Hellen Myezwa, Anne Söderlund

**Affiliations:** 1 Department of Physical Therapy, Faculty of Medicine, University of British Columbia, V6T 1Z3 Vancouver, Canada; 2 Department of Physical Therapy, New York University, New York, USA; 3 School of Physiotherapy, University of Otago, Dunedin, New Zealand; 4 Departamento de Fisioterapia, Universidade Federal de Pernambuco, Recife, Brazil; 5 Department of Physiotherapy, Witwatersrand University, Johannesburg, South Africa; 6 School of Health, Care and Social Welfare, Physiotherapy, Mälardalen University, Västerås, Sweden

**Keywords:** Health behaviors, Health professional entry-level curricula, Health promotion interventions, Lifestyle interventions, Lifestyle, Non-communicable diseases

## Abstract

**Background:**

To increase the global impact of health promotion related to non-communicable diseases, health professionals need evidence-based core competencies in health assessment and lifestyle behavior change. Assessment of health promotion curricula by health professional programs is a first step. Such program assessment is a means of 1. demonstrating collective commitment across health professionals to prevent non-communicable diseases; 2. addressing the knowledge translation gap between what is known about non-communicable diseases and their risk factors consistent with ‘best’ practice; and, 3. establishing core health-based competencies in the entry-level curricula of established health professions.

**Discussion:**

Consistent with the World Health Organization’s definition of health (i.e., physical, emotional and social wellbeing) and the Ottawa Charter, health promotion competencies are those that support health rather than reduce signs and symptoms primarily. A process algorithm to guide the implementation of health promotion competencies by health professionals is described. The algorithm outlines steps from the initial assessment of a patient’s/client’s health and the indications for health behavior change, to the determination of whether that health professional assumes primary responsibility for implementing health behavior change interventions or refers the patient/client to others.

An evidence-based template for assessment of the health promotion curriculum content of health professional education programs is outlined. It includes clinically-relevant behavior change theory; health assessment/examination tools; and health behavior change strategies/interventions that can be readily integrated into health professionals’ practices.

**Summary:**

Assessment of the curricula in health professional education programs with respect to health promotion competencies is a compelling and potentially cost-effective initial means of preventing and reversing non-communicable diseases. Learning evidence-based health promotion competencies within an inter-professional context would help students maximize use of non-pharmacologic/non-surgical approaches and the contribution of each member of the health team. Such a unified approach would lead patients/clients to expect their health professionals to assess their health and lifestyle practices, and empower and support them in achieving lifelong health. Benefits of such curriculum assessment include a basis for reflection and discussion within and across health professional programs that could impact the epidemic of non-communicable diseases globally, through inter-professional education and evidence-based practice related to health promotion.

## Background

Although non-communicable diseases (NCDs) have been described by the World Health Organization (WHO) as being largely preventable [1,2], priorities for action based on the foundation value of health promotion from the Ottawa Charter remain to be fully implemented [3]. These include strengthening structures and processes for health promotion, moving toward knowledge-based practice, and building a competent health promotion workforce through professional education that is responsive to societal priorities [4,5]. Scriven and Speller [3] have argued that advocacy at all levels of health delivery and care needs to continue to ensure that policy goals represent the principles of Ottawa..’. Consistent with this thrust, Mittelmark [6] described the need for setting an ethic agenda for health promotion based on dialogue resulting from the Ottawa Charter and more recently from the Bangkok Charter that established the cornerstone for health promotion. Others [7] have argued that health promotion must go beyond a narrow interpretation in the field and requires greater participation of people with respect to their health practices. We propose that health professionals can impact societal health more broadly by empowering their patients/clients with the universal practice of established core health promotion competencies that include the examination of health and health behaviors and implementation of strategies to modify these as needed.

The ultimate knowledge translation gap in health services delivery has been described as the one that exists between what is known unequivocally about the causal and contributory relationships between NCDs and lifestyle behavior, and the need to implement that knowledge into behavior change [8,9]. To address the global NCD epidemic, much has been documented about lifestyle behavior change to maximize health and reduce health risk though initiatives such as smoking cessation, and optimizing diet and physical activity. Although substantial health benefit can result from small changes in health behavior [10], assessing health-related lifestyle practices and effecting health behavior change constitute unique competencies. Comparable to the basis for drug prescription, they require systematic assessment of the patient’s/client’s needs and wants which may involve family and community, and implementation of one or more lifestyle-behavior change interventions, which up to now have not been systematically integrated within and across the curricula of health professional programs. Depending on identified needs and wants, patients/clients may require referral to one or more health professionals. The work of Blanchard and colleagues is sobering. It provides strong evidence for core health promotion competencies being practiced by health professionals [11] as opposed to vague advice such as ‘stop smoking’, ‘lose weight’, or ‘be more active’. These investigators reported that even when people receive the proverbial ‘wake-up call’, e.g., those who are diagnosed with cancer, their long-term adherence to healthy living recommendations is alarmingly poor.

The need for health promotion has been advocated across health professions including medicine, nursing, occupational therapy, pharmacy, and physical therapy, yet its implementation has lagged [12-17]. Examples of attempts to benchmark lifestyle behavior change curricula content have been made in some health professional programs, notwithstanding several challenges that challenge the validity and reliability of the data [18,19]. Although health behavior change is becoming an overarching priority consistent with population health systems, regardless of the particular health professional that a patient/client may be seeing, its practice remains fragmented and silo-ed [4,20]. A systematic approach based on core competencies that are shared within and across health professions would be a step toward bridging the gap between the value of health promotion and its systematic implementation into practice.

Key elements of health promotion practice include: systematic assessment of global health and health behaviors, people’s environmental and social contexts, and targeted interventions; accountability of the health professional and patient/client; and systematic follow-up. We propose that the effect of any health behavior change intervention that is initiated and/or supported by multiple health professionals will be augmented given the opportunity for the health message and interventions to be systematically and consistently reinforced.

Based on the WHO’s definition, health is not synonymous with the absence of signs and symptoms of a health condition or disease, but rather is a complete state of physical, mental and social wellbeing [21]. In the era of chronic NCDs, people may expect to live for many years with these conditions, if not a full life expectancy. The health backdrop of people with chronic health conditions is often not the primary focus of health services, which more often focuses on signs and symptoms. Maximizing health through health behavior change in its own right however warrants being a primary goal designed to: prevent the NCDs; reduce the signs and symptoms of these and other chronic conditions; and improve the outcomes of both non-pharmacologic/non-surgical interventions as well as pharmacologic/surgical interventions.

That ‘healthy living is simply good for you’ is a circuitous argument and one that is challenging to refute. Smith and Pell [22] deduced the value of such observational data based on a systematic review that addressed the value of parachutes to counter the negative effects of free fall gravitational force. We extend their logic regarding the validity of observation with respect to the benefits of parachute use, to the benefits of healthy living. We propose that assumptions can be made about healthy people and their reduced need for health services, and their augmented response to health services when needed compared with unhealthy people. Assumptions that can be made about healthy people include:

• Healthy people get sick less often.

• When they do, they are sick for less time, recovering faster with fewer complications.

• Healthy people need fewer biomedical procedures; take fewer drugs and need less surgery.

• When they need these, they need less medication for less time, and benefit from less invasive surgery.

• Healthy people place fewer social and economic demands on society.

• Healthy people stay in the workforce with less absenteeism, and long-term sick leave.

• Healthy people tend to leave their jobs for reasons other than sickness.

In a recent study [23], Wilson and colleagues showed that lifestyle modification was mentioned in fewer than half the studies in one major published report of an established Cochrane review of the effects of anti-hypertensive medication. Given that lifestyle modification is the established first-line best practice in the management of hypertension regardless of its severity or the presence of multiple co-morbidities [24], its omission in major drug trials raises critically-important questions about the degree to which research paradigms should reflect established best clinical practice. Masking the effects of lifestyle practices with sophisticated randomization methods may lead to undermining these powerful effects and failure to appreciate important interactions of lifestyle practices with medications. Conversely, a study of lifestyle practices on hypertension that did not consider the precise medication prescription of its participants would be considered methodologically flawed.

Given the powerful effects of healthy lifestyle practices, neglecting to assess lifestyle and prescribe healthy living practices to patients’/clients’ as systematically as evidence-supported medication by a highly qualified practitioner or prescribed surgery without the requirement for an equally evidence-based systematic assessment of health and health risks and evidence-based health behavior change strategies or interventions, is no longer justified in our view. In support of this position, we first define health promotion competencies. Then we outline a process for clinical decision making for a health professional related to patient/client health and risk factor assessment, criteria for referring to other health professionals, and the need for on-going support. Some simple tools are described that are universally accessible for the assessment of health and health risk and health behavior change strategies and interventions. These tools and strategies provide the basis of a template for program assessment of the curriculum content of health in health professional education programs. Assessment of this content constitutes one step toward establishing what health content is being included and the time devoted to it, in turn providing a basis for conversation within and among health professional entry-level programs about core competencies as well as more specialized competencies related to health by each specific established health profession.

### Defining health promotion competencies

Health is multidimensional, thus no single metric exists to assess it. The WHO has put forth the International Classification of Functioning, Disability and Health (ICF) that delineates three levels at which health status can be assessed and interventions targeted (level of body function and structure, activity, and participation) [25]. Global indices of health have become more common and include tools such as sickness/disability impact profiles, life satisfaction, wellbeing, and quality of life, which assess functional independence and social participation (subsumed within the contextual factors of the ICF, specifically, personal factors and environmental factors). Health promotion competencies are those that reflect the holistic construct of health in the ICF. Although reducing troublesome signs and symptoms may be the focus of care, attention to the comprehensive health needs of the patient/client requires advice about smoking reduction/cessation, basic nutrition, physical activity, sleep hygiene, and stress management; these being core health promotion competencies. Particularly, in chronic health conditions such as the NCDs, the degree to which such behavior change translates into improved activity and social participation is central to an individual’s overall health and wellbeing, given quality of life metrics are largely associated with the capacity to participate in one’s life’s roles. A healthy lifestyle is associated with better health outcomes with or without a health condition, and reduced risk for NCDs, thus healthy lifestyles warrant being advocated for all by health professionals. Such an approach may help address the social determinants of health however health policy is needed to effectively address population health outcomes.

### Assessment of health content of education curricula of health professional programs

Factors that inform contemporary health professional curricula have not been well described. Much of the content of journals on education in the health professions focuses on pedagogical issues, e-learning, and technology rather than examining how curriculum content should be established based on epidemiological, social, and economic considerations, and how curricula should respond to changing epidemiological trends. The content of health professional education curricula appears to reflect historic precedent rather than being informed by a coherent rationale based on epidemiology and societal priority. To make the point, if these curricula were to be designed for the first time today, in the current health climate, they are likely to look very different.

Evaluating health professional curricula is fraught with methodological challenges [18]. One challenge is who provides the information in terms of his or her knowledge and familiarity with the curriculum and his or her commitment to providing this information that can be time-consuming particularly in contemporary curricula committed to integrated and case-based learning. In addition, the quality of the data about the curriculum may be questionable due to a range of words and descriptors (general description of curriculum content or specifics), and the quality of specific content may be impacted by having to search horizontally and vertically across courses and years in the program. Such curricula evaluations have the potential for poor reliability and validity. To avoid these issues and improve the credibility of the data, program assessment may be a useful initial step toward reflection of programs regarding health promotion content, and comparison of the content of entry-level programs across health professions and arriving at consensus regarding what constitutes basic core health promotion competencies.

A benchmark of evidence-based health promotion content of entry-level curricula for health professional education programs augment program awareness and knowledge, and could facilitate development of a database to inform basic shared content across professions and individual content within a profession. As well, it could identify gaps that need to be addressed across and within programs. With such data available, discourse could then be initiated about minimal standards for inter-professional health promotion competencies including a process for inter-professional cross referral.

Table 1 itemizes health promotion curricula content at two levels (assessment/evaluation and health behavior change strategies and interventions) that could be considered core requirements for practice based on the health behavior change literature. The table includes a column for hours that a program includes in relation to each of these broad topics for each major health behavior, with respect to hours of theory, hours of practical application, and hours of clinical practice. To be integrated into the curricula of a health profession’s entry level education, health and health risk assessment/outcome evaluation, and evidence-based strategies and interventions to change several leading health behaviors, need to be taught as clinical competencies that warrant being included the management of most patients/clients.

**Table 1:** Assessment of health promotion competencies (assessment/evaluation and health behavior change strategies and interventions) for entry-level health professionals in their program curricula

**Health behavior**	**Assessment and outcome evaluation**	**Overall hours Theory: Practical: Clinical**	**Health behavior change strategies and interventions**	**Overall hours Theory: Practical: Clinical**
Smoking	**Goal: Non smoker**		Readiness-to-change stage-based interventions	
	Non smoker		Pre-contemplative stage	
			→ 5 R’s (Relevance, Risks,	
	Ever smoked, if so, how much for how long Number of quit attempts		Rewards, Roadblocks, Repetition)	
			Contemplative/preparation/action stages	
			→ 5 A’s (Ask, Advise, Assess, Assist, Arrange)	
	Smoker: how much for how long		Formal established training program in smoking cessation, e.g., http://www.quit.org.nz/94/helping-others-quit/health-professionals	
	Number of quit attempts			
	Equivalent of ‘The Why Test’ to establish motivation for smoking		Advice, e.g., cutting back, setting a quit date, garnering social support, goal setting, developing competing interests, e.g., exercise	
	Readiness to quit			
			Nicotine replacement therapy	
			Counseling strategies:	
			Motivational interviewing	
			Cognitive behavior therapy	
			Acceptance commitment therapy	
			Other: e.g., quit blogs	
Nutrition	**Goal: Healthy body mass and body fat, and healthy lean tissue**		Readiness-to-change stage-based interventions	
			Pre-contemplative stage	
			→ 5 R’s	
	Body mass index		Contemplative/preparation/action stages	
	Waist-hip ratio			
	Servings of vegetables daily		→ 5 A’s	
	Goal: >5 A-Day		Counseling strategies:	
	Servings of fruit daily		Motivational interviewing	
	Whole grains servings daily		Cognitive behavior therapy	
			Acceptance commitment therapy	
	Low red meat and processed meat consumption			
	Readiness to eat more healthily		Other:	
Activity and exercise	**Goal: ↓ Sedentary activity**		Readiness-to-change stage-based interventions	
			Pre-contemplative stage	
	**↑ Regular physical activity daily and structured exercise 3-5 x/wk**		→ 5 R’s	
			Contemplative/preparation/action stages	
			→ 5 A’s	
	Walks around hourly during periods of prolonged sitting		Counseling strategies:	
			Motivational interviewing	
			Cognitive behavior therapy	
			Acceptance commitment therapy	
	Hours of prolonged sitting work day		Other:	
	Hours of regular physical activity			
	Moderately-intense activity			
	Regular structured exercise			
	Aerobic			
	Strength			
	Yoga/tai chi			
	Readiness to be more active			
Sleep	**Goal: 7-9 h/night**		Readiness-to-change stage-based interventions	
	Average number of hours		Pre-contemplative stage	
			→ 5 R’s	
	Average number of times up at night		Contemplative/preparation/action stages	
			→ 5 A’s	
	Quality of sleep overall (0 =worst to 10=best)			
			Counseling strategies:	
	Readiness to improve sleep quality and quantity		Motivational interviewing	
			Cognitive behavior therapy	
			Acceptance commitment therapy	
			Other:	
Mental health (anxiety and stress)	**Goal: Feels unhurried and can manage stress most days**		Readiness-to-change stage-based interventions	
			Pre-contemplative stage	
			→ 5 R’s	
	Daily irritations		Contemplative/preparation/action stages	
	Life challenges Holmes Rahe Stress test		→ 5 A’s	
			Counseling strategies:	
	Readiness to reduce stress		Motivational interviewing	
			Cognitive behavior therapy	
			Acceptance commitment therapy	
			Other:	

The columns titled Overall Hours (Theory: Practical: Clinical) are where the number of hours are entered by a given health professional program for each topic (assessment/outcome evaluation and each health behavior change strategy and intervention). Hours are categorized as theory, practical, and clinical.General tools to assess Global Health: e.g., Health Improvement Card (Figure 2).Template of a tool to assess non-communicable disease risk: e.g., type 2 diabetes mellitus (CANRISK) (Figure 3).

## Discussion

Based on recent reports in The Lancet and from the WHO, health professionals continue to practice largely in silos [4,20]. They have focused largely on their unique competencies that define them professionally and limited competencies that could be performed by others. Inter-professional health service delivery including shared goals is being strongly advocated. Inter-professional health service delivery is hallmarked by a commitment to evidence-based practice and commonalities of approaches to service delivery. No benchmarks exist for health promotion content in the entry-level of health professional education curricula. The notion of focal areas to support international co-operation of global stakeholders in health promotion has been raised by Magnussen who argued such collaboration would result in greater impact [26].

Most health professions are committed to evidence-based practice including knowledge translation and integration. The elements of evidence-based health promotion competences include a process for assessment and intervention that is common across health professionals, thus facilitating the integration and implementation of such clinically-relevant tools into their practices.

### Process: Health promotion clinical decision making

**Figure 1:**
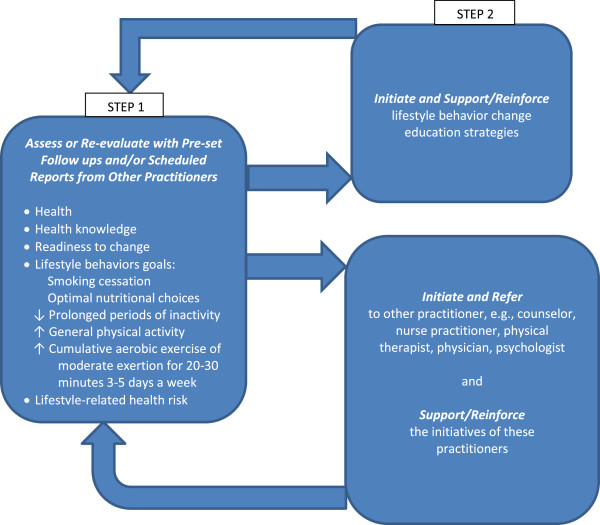
**Steps in the decision making process for health professionals to augment their patient/client outcomes by initiating and/or supporting lifestyle-related health behavior changes.** Source: adapted from Dean et al. 2012 [8].

Inter-professional health promotion competencies require a shared process and shared context to guide their implementation. An algorithm that outlines the steps in this process is shown in Figure 1. Each health professional needs to assess the patients’/clients’ health, lifestyle practices, presence of or risk for the NCDs and their risk factors, and readiness to change health behaviors. Readiness to change includes personal readiness, the reliance on social support and family for such change, and the physical environment to support health behavior change. Based on these assessments, each health professional would then determine what health behavior change strategies and interventions are within their competency and determine if they take a primary role in effecting a given health behavior change or they refer to one or more other health professionals. Regardless of whether they intervene or refer, health professionals must be responsible for appropriate follow up to assure a life-long positive change. Timely, re-evaluation may indicate refinement or revision of the program or whether re-assessment of the role of other health professionals is needed.

Competencies that could be shared inter-professionally both during education and in practice, fall into two categories (Table 1): 1) health assessment and outcome evaluation tools and 2) health behavior strategies and interventions.

### Competencies: Health assessment and outcome evaluation tools

No single test or measure exists to assess health. Global health assessment tools include those for sickness impact, life satisfaction, wellbeing, and quality of life. The use of such tools cross references with a comprehensive health profile within the framework of the ICF. The ICF provides a framework for assessing health at levels other than only functional and structural limitations, namely, activity and social participation.

**Figure 2:**
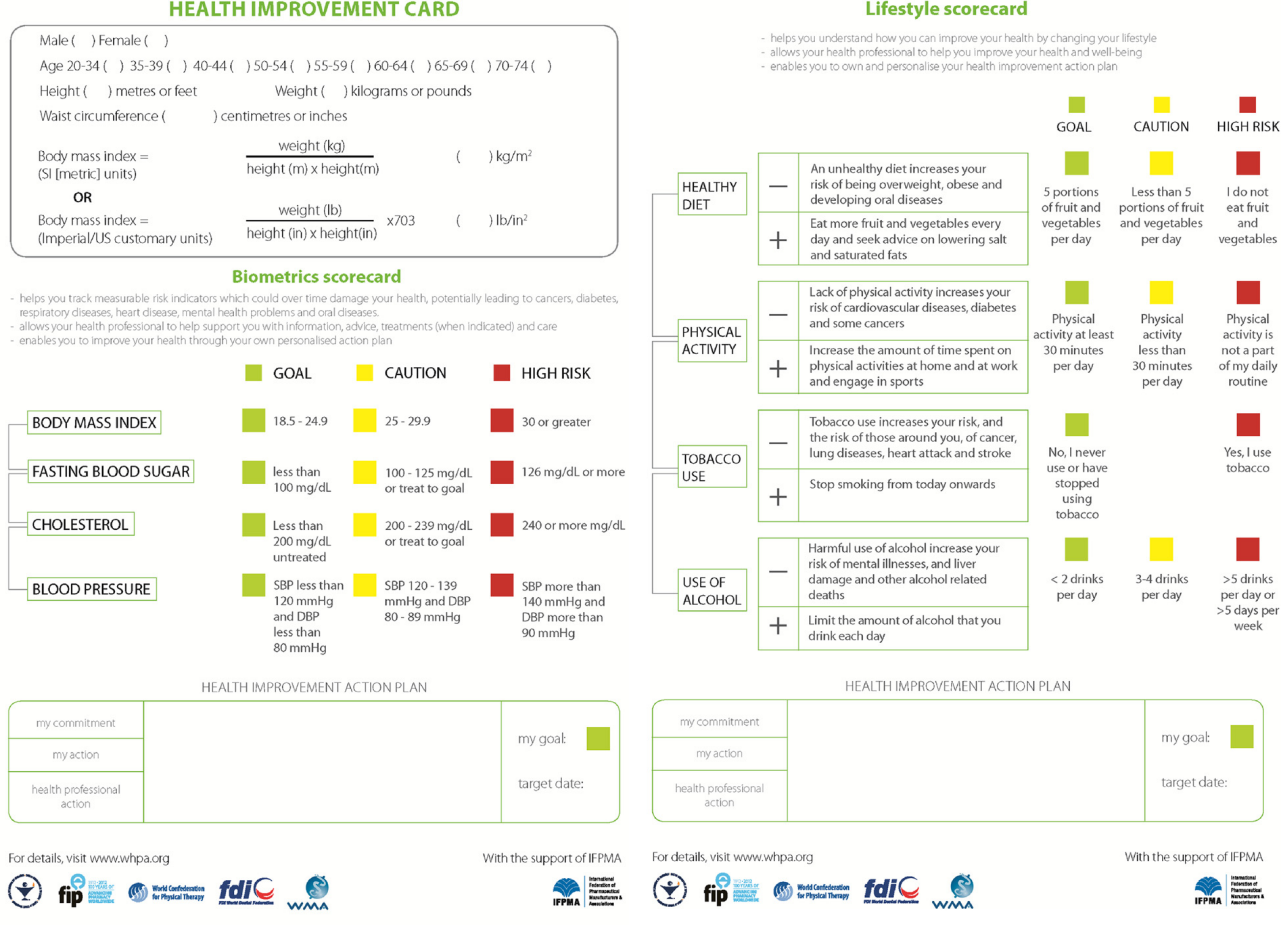
**Health Improvement Card.** Source: Health Improvement Card. Reprinted with permission from the World Health Professionals Alliance, 2014. http://www.ifpma.org/fileadmin/content/Publication/2011/ncd_Health-Improvement-Card_web-1.pdf.

In 2012, the World Health Professions Alliance, a group of six leading health professional organizations representing over 26 million health professionals [27], published the Health Improvement Card [28] (Figure 2) so health professionals can readily assess a patient’s/client’s health and make recommendations to improve his or her health.

Health professionals need competency in the assessment and outcome evaluation of several health behaviors related to the NCDs and their risk factors. Most notably, these include the status of a patient/client with respect to tobacco use; harmful use of alcohol; unhealthy diet; overweight/obesity; prolonged periods of sitting; insufficient physical activity; disturbed sleep; and unmanageable stress; in addition to objective measures including raised blood pressure, raised blood sugar, and raised cholesterol. Table 1 lists some tools that can be used to assess these.

**Figure 3:**
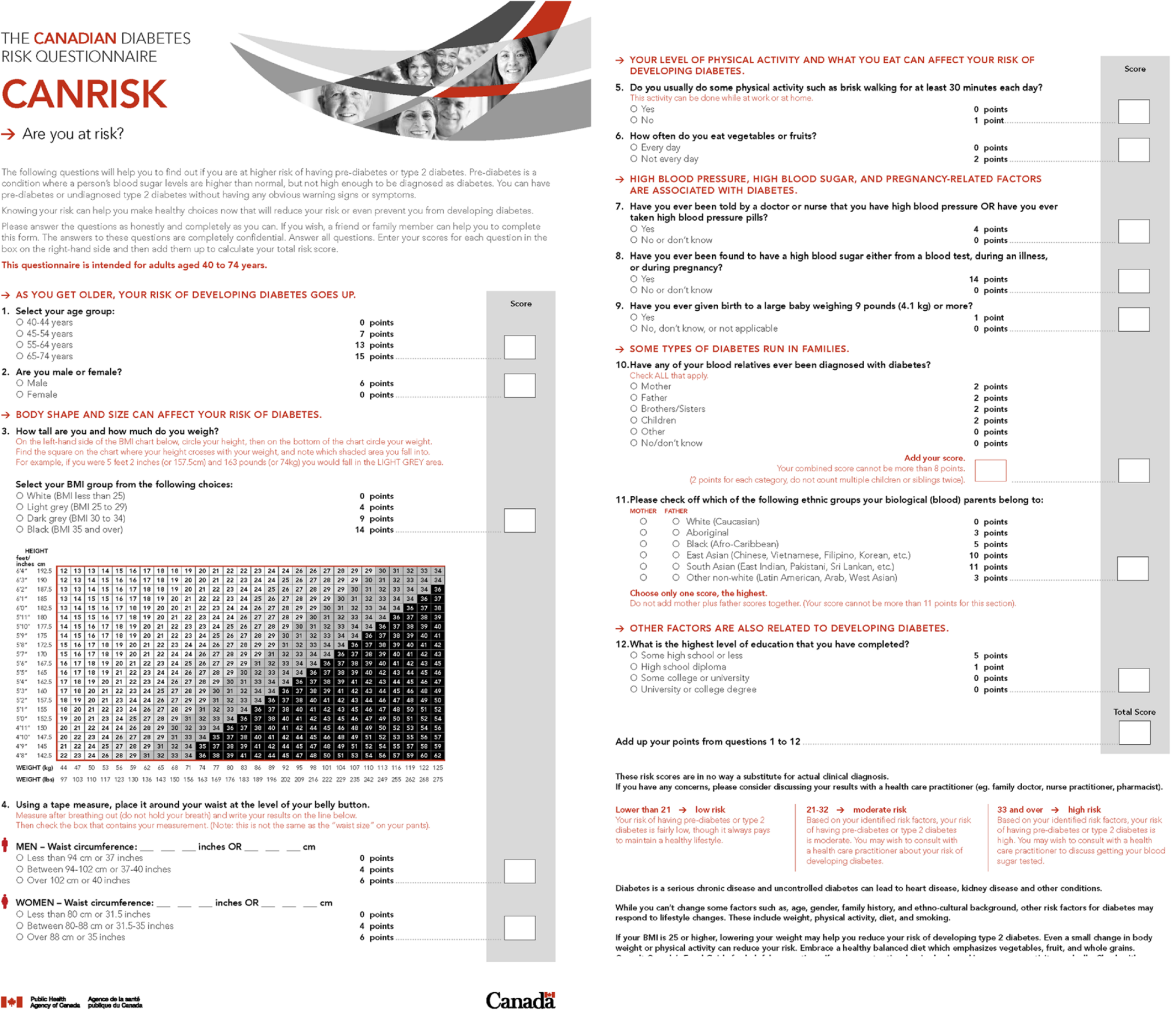
**Prototype of a lifestyle-related health risk assessment tool: CANRISK.** Source: *©* All rights reserved*.* Public Health Agency of Canada. Reproduced with permission from the Minister of Health, 2014.

Valid and reliable lifestyle behavior risk factor assessment tools do exist. It would be neither time nor resource effective for health professionals however to administer risk assessment questionnaires for each NCD and each risk factor. Risk factors for these conditions have commonalities therefore selection of one may help to provide a risk factor assessment for lifestyle-related conditions in general. One comprehensive form that may serve as a template is the short (12 questions) type 2 diabetes mellitus risk factor assessment form entitled CANRISK [29] (Figure 3). Many of the questions reflect risk for other lifestyle-related conditions therefore if a generic risk factor assessment tool were to be used, a rationale could be made for CANRISK.

### Competencies: Health behavior change strategies and interventions

Competency in several health behavior change strategies and interventions are needed by health professionals in areas that address the NCDs, including smoking cessation; healthy nutrition; weight loss; reduced sedentary behavior and increased physical activity; optimal sleep and reduction in unmanageable stress. Table 1 itemizes evidence-based behavior change strategies and interventions related to the lifestyle health practices of patients/clients that can be readily integrated into the busy, time- and resource-constrained practices of health professionals.

## Summary

Health assessment and effective health behavior change are unique health promotion competencies with a strong evidence base. Such competencies need to be viewed with the same rigor as competencies required for impairment examination to prescribe exercise, functional training, or medications. A template has been presented for a program of assessment of health promotion competencies in the curricula of health professional programs. Such assessment is a first step toward dialogue regarding common health promotion competencies within and across health professions. By unifying the approach of health professionals to the NCDs and their risk factors that includes health and lifestyle practice assessments and knowledge of effective health behavior change strategies and interventions, inter-professional ‘best practice’ can be achieved. A united front of health professions in the eyes of patients/clients in addressing these burdensome NCDs and their risk factors would be a major step forward in addressing these conditions globally. It would also highlight through consistent personal messaging to the public that their health professionals are committed to best practice and reversing the NCD epidemic through cost-effective measures. Such inter-professional health promotion competencies emphasize to the public that non pharmacological interventions are as important, if not more important in many cases, than invasive (drug and surgical) interventions in the management of the NCDs.

## Competing interests

The authors declare that they have no competing interests.

## Authors’ contributions

ED, HM, AD, MS have contributed to this debate based on participation in The First and Second Physical Therapy Summits on Global Health (Vancouver, 2007 and Amsterdam 2011), and planning of the Third Physical Therapy Summit on Global Health (to convene in Singapore 2015). MM has contributed through position statements of the World Confederation for Physical Therapy in her capacity as president of the organization, and through her contribution to framing and editing the ideas and their development. AS has contributed through scholarly collaboration with ED related to her areas of expertise in behavioral medicine and related clinical decision making. In addition, AS’s broad-based perspectives and insights derive from her role as Editor-in-Chief, European Journal of Physiotherapy. All authors read and approved the final manuscript.

## Pre-publication history

The pre-publication history for this paper can be accessed here:

http://www.biomedcentral.com/1471-2458/14/717/prepub
